# Photoconductivity Relaxation Mechanisms of InGaAs/GaAs Quantum Dot Chain Structures

**DOI:** 10.1186/s11671-017-1954-7

**Published:** 2017-03-09

**Authors:** Serhiy V. Kondratenko, Sviatoslav A. Iliash, Oleg V. Vakulenko, Yuriy I. Mazur, Mourad Benamara, Euclydes Marega, Gregory J. Salamo

**Affiliations:** 1 Department of Physics, Taras Shevchenko National University of Kyiv, 64 Volodymyrs’ka St., Kyiv, 01601 Ukraine; 2 Institute for Nanoscience and Engineering, University of Arkansas, Fayetteville, AR 72701 USA; 3 Instituto de Fisica de São Carlos, Universidade de São Paulo, CP. 369, São Carlos, SP 13560-970 Brazil

**Keywords:** Quantum dot chain, InAs/InGaAs, Nanostructure, Semiconductor, Photoluminescence, Photoconductivity recombination, Quantum-size state

## Abstract

An experimental study of the photoconductivity time decay in InGaAs/GaAs quantum dot chain structures is reported. Different photoconductivity relaxations resulting from spectrally selecting photoexcitation of InGaAs QWR or QDs as well as GaAs spacers were measured. The photoconductivity relaxation after excitation of 650 nm follows a stretched exponent with decay constant dependent on morphology of InGaAs epitaxial layers. Kinetics with 980 nm excitation are successfully described by equation that takes into account the linear recombination involving Shockley–Read centers in the GaAs spacers and bimolecular recombination via quantum-size states of InGaAs QWRs or QDs.

## Background

Optical properties and characteristics of charge carrier’s transport in quantum-dimensional heterostructures based on semiconductor III–V materials are widely studied by scientists in recent years [1]. Such an interest caused by self-assembled (In,Ga)As quantum dots (QDs) and quantum wires (QWRs) are perspective candidates for application in novel electronic and optoelectronic systems, e.g., semiconductor lasers [2], infrared photodetectors [3], and solar cells [4, 5]. This is possible due to quantum confinement, energy disorder, complex electronic spectra of the system, and coexistence of 2D and 1D states. These factors are important for enhanced electrical conductivity, as well as 1D states and defect states that can play a role in the band bending, trapping, and recombination at the InGaAs/GaAs interface [6]. Besides the states of different dimensionalities available for transport, the system’s photoconductivity is further complicated due to ionized defects, impurities, or trapped charges in the vicinity of QD inducing local electric fields that can reach a strength of several 10 kV/cm. Their spatial scale and amplitude determined surface density of nanoscale objects, size variations and component composition, mechanical strain field, random spatial distribution, and population of localized states [7]. Moreover, each QD is exposed to a different electric field having strongly influenced its electronic structure [8], carrier relaxation [9], transport mechanisms, and recombination of photoexcited charge carriers.

In this paper, we report the experimental studies of the photoconductivity relaxation in InGaAs/GaAs quantum dot chain structures with different inter-dot distances by varying the excitation photon energy. Investigations of photoconductivity time constants as well as identification of decay function allow us to understand the mechanism of the charge carrier recombination via quantum states of the InGaAs or deep level traps.

## Methods

Heterostructures with InGaAs/GaAs quantum dot chains were grown by MBE on GaAs (100) semi-insulating substrates. The structures consisted of 15 layers of In_x_Ga_1–x_As QDs separated by 60 monolayer (ML) thick GaAs spacer layers. Two different samples with In_x_Ga_1–x_As coverages of 5.7 and 15.5 ML and In compositions of *x* = 0.5 and 0.3, respectively, were grown. The first kind of samples was formed QDs, while the second one contains QWRs. The growth procedure of these heterostructures was described in detail in Refs. [7, 10, 11]. All samples were terminated with a final uncapped layer of InGaAs, grown with the same composition and coverage as the underlying InGaAs layers in order to match the nanostructures and examine the configuration by atomic force microscopy. The HRTEM images were directly recorded on a Gatan 2K CCD camera with Digital Micrograph acquisition software.

Ohmic contacts were formed by annealing indium on the surface at 420 °C in N_2_ ambient, such that conductivity measurements can be performed for in-plane, lateral transport.

**Fig. 1:**
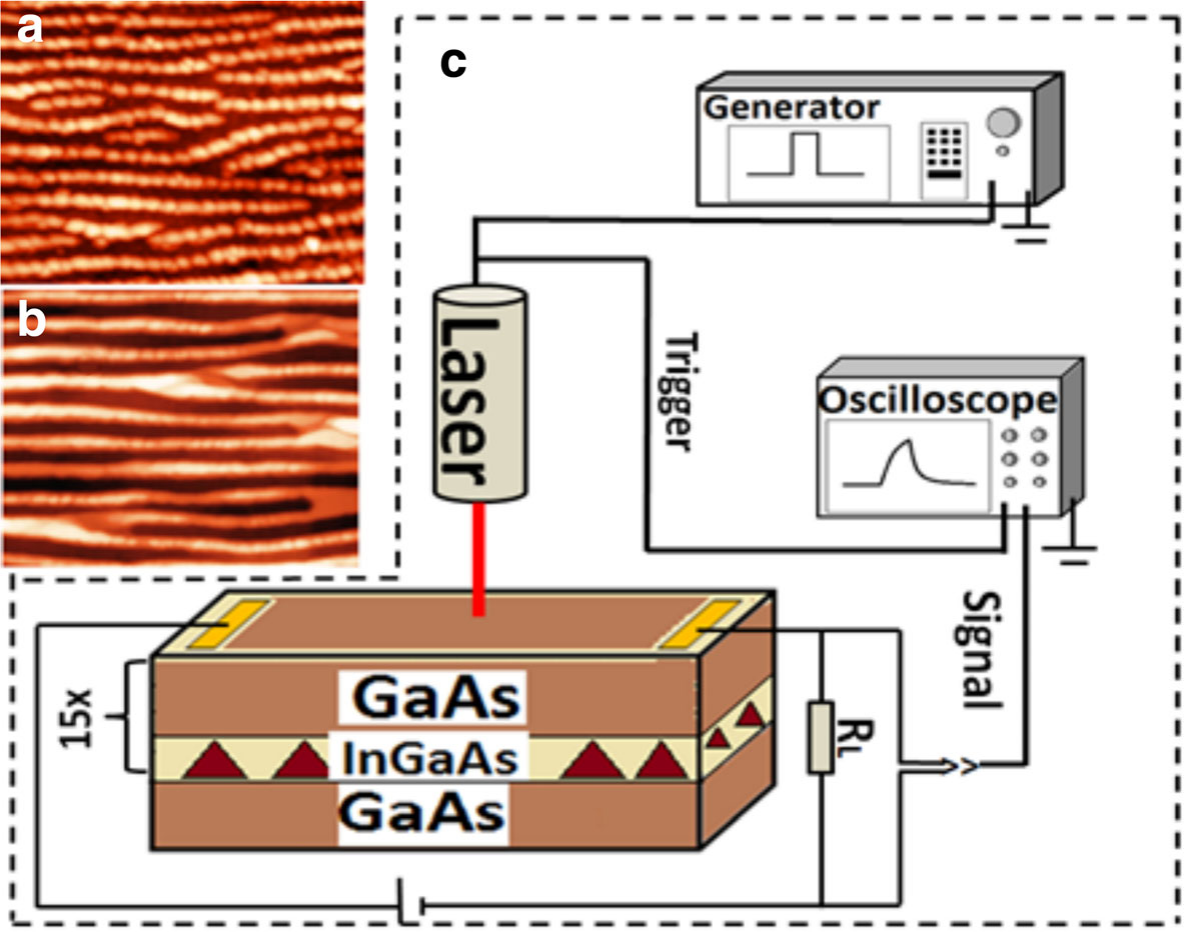
(Color online) AFM images of surface with dot-chain (**a**) and QWRs (**b**). Scheme of the PC trancient measurement (**c**)

The current temporal dependencies were recorded on a Siglent 70-MHz-bandwidth and 1 MΩ input impedance digital oscilloscope with a low-noise amplifier (AD8138) that measured a voltage signal drop across a series load resistance of 50 Ω (see Fig. 1). All measurements were carried out by applying a constant bias voltage of 50 mV to the sample along dot chains, parallel to the [0–11] crystallographic direction. The photoconductivity (PC) was determined from the total current under illumination after subtracting the dark current. The samples were excited using an optical pulse generated by laser diodes with emission at 650 and 980 nm with a pulse width of ~60 μs with rise and decay times of ~10 ns. The shape of the PC temporal dependencies does not depend on load resistance that gives reason to exclude the effects related to RC-time constants.

Photoluminescence (PL) measurements were carried out over a wide temperature range in order to better understand the effect of dimensionality and morphology on the electrical and optical properties of InGaAs/GaAs dot chain heterostructures. For excitation, the 532 nm line of a frequency, doubled Nd:YAG laser, was focused to a ~20-μm diameter spot at the sample. The samples were mounted in a variable temperature, 10–300 K, closed-cycle helium cryostat, and the PL signal from the sample was dispersed by a monochromator and detected by a liquid nitrogen cooled OMA V: InGaAs photodiode detector array.

## Results and Discussion

### AFM and TEM Measurements

**Fig. 2:**
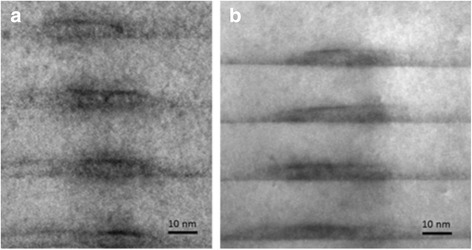
(Color online) Cross-sectional TEM images of InGaAs/GaAs QWR (**a**) and quantum dot-chain (**b**) structure along the [110] direction

A topographic AFM image of InGaAs/GaAs sample shown in Fig. 1a reveals long QD chains aligned along the [0–11] direction separated by 74 ± 4 nm. The average distance between the centers of the QDs along the chains (the [0–11] crystallographic direction) is about 65 nm, in sample with quantum dot chains. The QD sizes in this sample depend on the InGaAs coverage and composition. Typically, the QD shape is slightly elongated along the chain direction of [0–11] [12]. Such unique growth morphologies are mediated by the asymmetric surface diffusivities on the GaAs (001) surface. Figure 2 shows a cross-sectional high resolution TEM image of InGaAs QD chains (b) and QWR (a) in the GaAs matrix. The perfect crystal planes for the GaAs spacer layers were observed. As was shown earlier [13] by geometrical phase analysis of HRTEM images, the non-uniform elastic stress relaxation mainly occurs at the tip of the dot and that the underlying GaAs layer is under tension.

### Photocurrent and Photoluminescence Spectroscopy

**Fig. 3:**
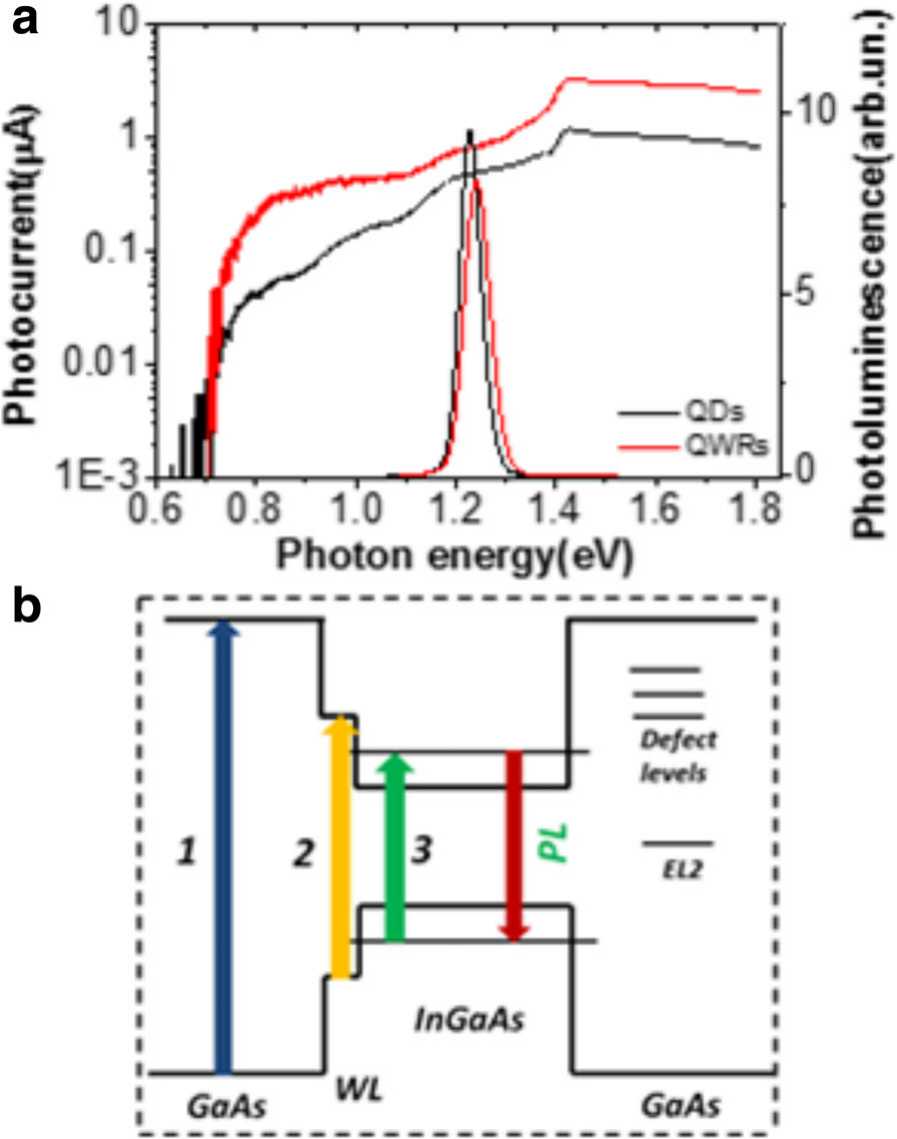
(Color online) **a** PL and PC spectra of InGaAs/GaAs heterostructures with QWR and QD, at 290 K. **b** Electronic transitions in InGaAs/GaAs heterostructures: (*1*) GaAs, (*2*) WL, and (*3*) InGaAs dimensional quantum states

The PL and PC spectroscopy shows the presence of several electronic transitions in the studied structures. The PL spectra of samples, measured at 290 K using power excitation about ~0.04 mW/cm^2^, are shown in Fig. 3a. This luminescence is associated with radiative transitions between quantum states of InGaAs [14]. Also, Fig. 3a shows the spectral dependence of photoconductivity measured with applied bias voltage of 50 mV in the energy range *hv* = 0.6–1.8 eV at 290 K. Interband transitions (arrow 3 in Fig. 3b) involving dimensional quantum states of InGaAs start contribute to the photocurrent from *hv* ≈ 1.11 and 1.12 eV for samples with QDs and QWRs, respectively. This threshold energy is determined based on the spectral position of the minimum radius of curvature.

Interband transitions in the wetting layer contribute to the PC spectra from 1.39 eV. At photon energies less than the band gap of nanostructures (~1.06 eV at 290 K), observational PC component is not associated with interband transitions. This photoconductivity component is associated with transitions involving deep levels in the InGaAs or GaAs bandgap. Since 0.74 eV, PC increase causes electronic transitions involving EL2 center in GaAs [15, 16]. This deep defect EL2 is well-known donor, who may have several different ionized charge states: EL2^0^, EL2^+^, and EL2^++^. Transitions from deep levels, E_c_–0,74 eV, close to the middle of the GaAs band gap [15], GaAs to conduction band generate free electrons and change the state of centers charge, for example changing such as EL2^0^ → EL2^+^. At the same time, electron transitions from the GaAs valence band to E_v_ + 0.67 eV and E_v_ + 0.47 eV (at 82 K) EL2^+^ and EL2^++^ centers, lead to the appearance of free holes due to changes EL2^+^ → EL2^0^ and EL2^++^ → EL2^+^, respectively [16]. The scheme of electronic transitions in InGaAs/GaAs heterostructures, obtained by PL and PC spectroscopy, is shown in Fig. 3b. It is shown that the contribution in photoconductivity from 0.74 to ~1.1 eV due to transitions across EL2 level is observed for both samples with QD and QWR. We can conclude the role of EL2 defects for photoconductivity typical for samples with varying morphology (QD and QWR).

### Photoconductivity Transients

**Fig. 4:**
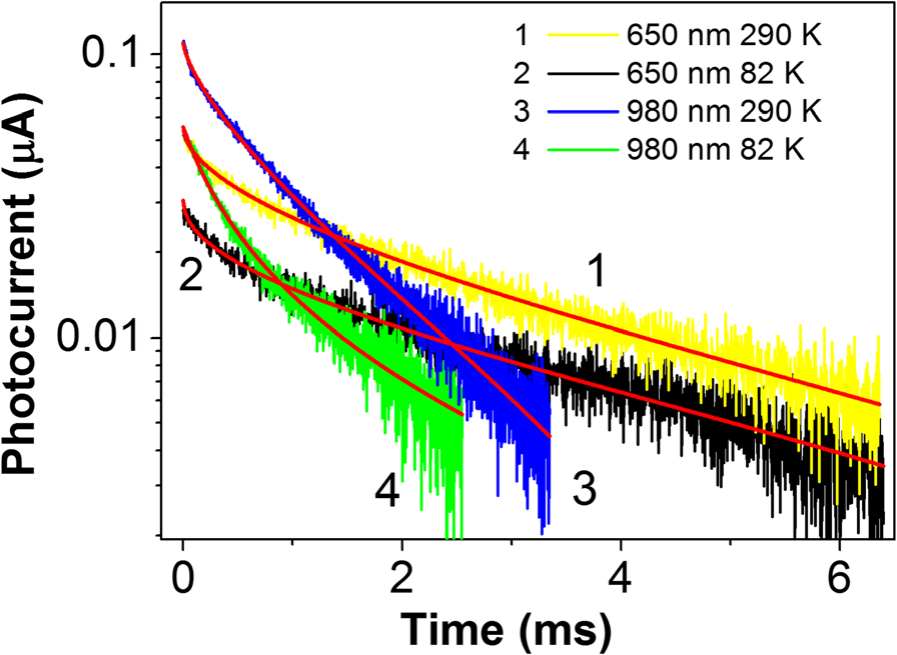
(Color online) PC decay kinetics for InGaAs/GaAs sample with QWR at temperatures 82 and 290 K and probe wavelength of 980 and 650 nm. The calculated stretched exponential curve with *τ*_*dec*_ = 3.3 ± 0.4 ms, β = 0.48 ± 0.02 at 82 К and *τ*_*dec*_ = 2.1 ± 0.1 ms, β = 0.55 ± 0.02 at 290 К are shown (*curve 1* and *curve 2*). For probe wavelength of 980 nm, the calculated exponential curve using function (2) with *τ*_*dec*_ = 1.59 ± 0.02 ms at 290 K (*curve 3*) and *τ*_*dec*_ = 3.7 ± 0.2 ms at 82 K (*curve 4*) are shown

Figure 4 shows photoconductivity relaxation kinetics for the sample with InGaAs QWR at temperatures 82 and 290 K. The structures are excited by light pulses with wavelength of 980 and 650 nm. Faster relaxation appeared at λ = 980 nm when the electron–hole pairs excited mainly by the band-to-band transitions in the InGaAs quantum wires and in the wetting layer, at the same time, when the excitation was λ = 650 nm, the main contribution to the photoconductivity gave interband transitions in the GaAs spacers. The difference in the relaxation curves was not observed, when the PC studied either along the chains or perpendicular direction due to the existence of an isotropic channel transport of photoexcited carriers in GaAs spacer layers and/or wetting layer.

**Fig. 5:**
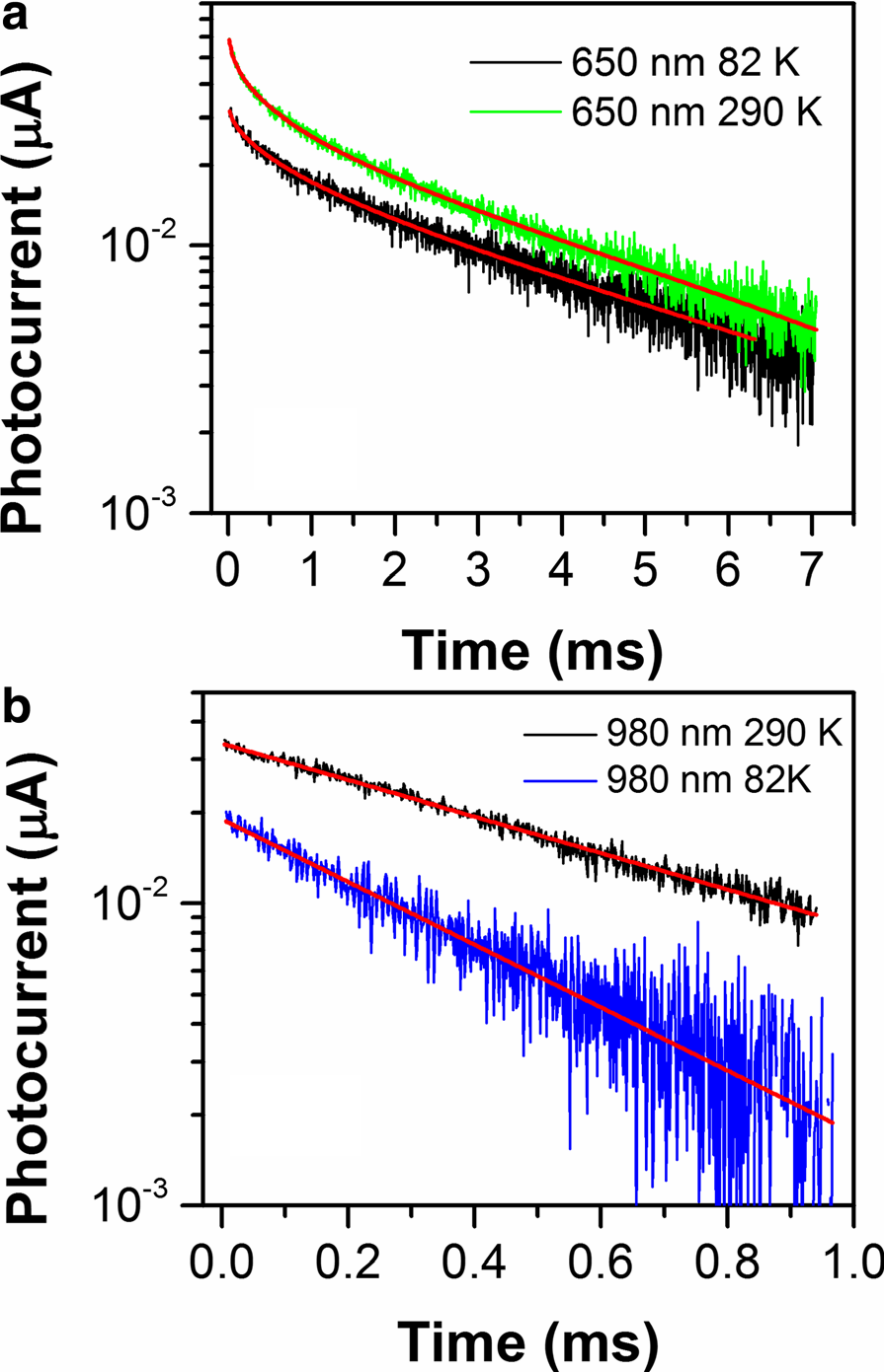
(Color online) PC decay kinetics for InGaAs/GaAs sample with QD at 82 and 290 K. Calculated stretched exponential curve for probe wavelength of 650 nm with *τ*_*dec*_ = 2.7 ± 0.2 ms, β = 0.53 ± 0.02 at 82 К and *τ*_*dec*_ = 1.7 ± 0.1 ms, β = 0.38 ± 0.01 at 290 К are shown (**a**). For probe wavelength of 980 nm, calculated exponential curve with *τ*_*dec*_ = 0.42 ± 0.02 ms at 82 K and 0.72 ± 0.02 ms at 290 K are shown (**b**)

Photoconductivity relaxation after excitation by 650 nm for both type of samples either InGaAs QDs or QWR follows a stretched exponential function:

1 $$ \varDelta G(t)\sim \exp \left[-{\left( t/{\tau}_{dec}\right)}^{\beta}\right], $$

where 0 < β < 1—ideality factor and *τ*_*dec*_—the time constant. Such a law is observed in the study of PL and PC relaxation in nonequilibrium systems [17]. We fitted the experimental curves for structure with QWR with *τ*_*dec*_ = 3.3 ± 0.4 ms, β = 0.48 ± 0.02 at 82 К and *τ*_*dec*_ = 2.1 ± 0.1 ms, β = 0.55 ± 0.02 at 290 К (see Fig. 4). The error to these fit is <0.4%, indicating good agreement for the stretched exponential form. For a sample with quantum dot chains photoconductivity relaxation curves after excitation with 650 nm, laser pulse (see Fig. 5a) were also fitted by a stretched exponential function with *τ*_*dec*_ = 2.7 ± 0.2 ms, β = 0.53 ± 0.02 at 82 К and *τ*_*dec*_ = 1.7 ± 0.1 ms, β = 0.38 ± 0.01 at 290 К. The observed photoconductivity relaxation scales, ~ms, are much larger compared to photoluminescence relaxation times ~ns [18] due to defect states in the GaAs spacers. The presence of defects cannot be excluded for both the InGaAs dots or the wetting layer also. In this case, they are additional recombination channels for electron–hole pairs localized by the InGaAs quantum dots, which reduces the photoluminescence quantum efficiency and decreases their lifetime, however, are not responsible for prolonged lifetime of free carriers contributing to the photoconductivity. Exclusively, the slow electron traps and Shockley–Read recombination centers in the dot’s surroundings with variety of activation energies and emission/capture rates exchanging by electrons with conductivity band only cause a considerable delaying of the PC relaxation as compared with typical intrinsic recombination times.

However, the photoconductivity relaxation after 980 nm excitation is not followed by a stretched exponent. For structures with QWR, the PC decay curves were fitted by function [19]:

2 $$ {I}_{PC}\sim \frac{ \exp \left(-\frac{t}{\tau_{dec}}\right)}{1+ A*\left(1- \exp \left(-\frac{t}{\tau_{dec}}\right)\right)}, $$

which is the solution of the kinetic equation considering both the linear via Shockley–Read recombination centers in the GaAs spacer layers and the bimolecular recombination via quantum states of the InGaAs. In general, decay kinetics of the photoexcited electrons is described by the equation:

3 $$ \frac{\partial n}{\partial t}=- an- b{n}^2, $$

where *n*—electron concentration, *a*—probability of linear recombination, and *b*—bimolecular recombination probability. Solution of Eq. (3) can be expressed as:

4 $$ \frac{n}{n_{\infty }}=\left(1+\gamma {n}_{\infty}\right) \exp \left(- at\right)-\gamma {n}_{\infty }, $$

where *n*_∞_—the stationary equilibrium concentration of electrons, *γ* = *b*/*a*. Following from formula (4), when *at* < < 1, the decay curve follows a hyperbolic law *n*, if *at* > > 1, the decay is exponential. Crossover to “stretched” kinetics is produced by substitution of *at* → (*t*/*τ*_dec_)^*β*^. As a result, the temporal dependence of electron concentration is

5 $$ \frac{n}{n_{\infty }}=\left(1+\gamma {n}_{\infty}\right) \exp \left(-{\left( t/{\tau}_{\mathrm{dec}}\right)}^{\beta}\right)-\gamma {n}_{\infty }. $$

In practice, we used the formula (2), implying that *I*_*PC*_ ~ *n* and *A* ~ *γn*_∞_. The resulting function with an allometric index (*t*/*τ*_dec_)^*β*^ is universal: if (*t*/*τ*_dec_)^*β*^ ≪1, the expression (5) becomes the stretched hyperbole; otherwise, when (*t*/*τ*_dec_)^*β*^ ≫1, we get the stretched exponent function.

Figure 4 shows experimental data for the structure with QWRs. The curves were fitted with a non-exponential function (2) with decay constants *τ*_*dec*_ = 3.7 ± 0.2 ms and *τ*_*dec*_ = 1.59 ± 0.02 ms at temperatures of 82 and 290 K, respectively.

Photoconductivity relaxation of structure with QDs excited with 980 nm excitation was found to be significantly faster compared with the structures with QWRs and well described by a single exponential function (see Fig. 5b). The decay constants *τ*_*dec*_ were 0.42 ± 0.02 ms and 0.72 ± 0.02 ms at temperatures of 82 and 290 K, respectively.

Thereby, depending on wavelength of excitation, the photoconductivity relaxations were described either a stretched exponential function (1) at excitation with λ = 650 nm or a function (2) at excitation with λ = 980 nm.

Let us discuss the possible reasons for this difference. Stretched exponential kinetics originated from linear recombination of nonequilibrium charge involving different Shockley–Read centers, which energy dispersion is caused by structural disorder. In more details, the excitation with 650 nm creates electron–hole pairs in both the GaAs spacers and the InGaAs, which causes photoconductivity. Upon switching off the excitation, those free electrons in the conductivity channels recombine with holes and the conductivity drops. The Shockley–Read centers play a key role in recombination slowdown of electron–hole pairs in GaAs spacers due to carrier trapping by deep levels. As noted in the works [7, 8, 20], the nearest surrounding of InGaAs quantum dots is characterized by the presence of some defect levels with different energies and fluctuations of the energy band edge potential in the GaAs spacers and deep levels position in the bandgap of GaAs, most likely, due to strain fields and fluctuations of the composition (concentrations of indium and gallium atoms) in the epitaxial planes. There might be different paths (e.g., recombination through different defects) for different carriers, characterized by different mono-exponential functions. Their total impact on the recombination processes of nonequilibrium charge carriers, in fact, leads to the observation of the multi-exponential photoconductivity relaxation, which are usually fitted by Kohlrausch stretched exponential function [17]. Under such conditions, time constant, *τ*_*dec*_, is the ensemble average recombination time, while the ideality factor β is associated with the energy dispersion.

The PC relaxation appeared slower for structures with QWR, indicating a longer lifetime for charge carriers photoexcited in the GaAs surrounding of InGaAs. The obtained results are in a good agreement with PC spectroscopy data (see Fig. 3a), where the photocurrent was higher for heterostructures with QWRs, i.e., samples with smaller areas of InGaAs/GaAs interface. Sample with QD shows the lowest photoconductivity, with higher mechanical stresses and larger area of interfaces than QWR. This leads to a higher concentration of defects, which act as recombination centers that reduce photoconductivity and decrease lifetime of charge carriers for both the 650 and 980 nm excitations.

While filling different types of electron traps in the dot surrounding was found to be the origin of a multi-exponential photoconductivity relaxation involving levels of Shockley–Read centers in the GaAs spacers, the InGaAs nanostructures are responsible for observation of the lower τ in the case of their selective excitation with wavelength of 980 nm. Moreover, observation of the effective radiative recombination involving InGaAs quantum-sized states and PC nonlinearities [21] gives further evidence of strong impact of InGaAs on PC relaxation. This was taken into account for formula (5), which has received confirmation during PC kinetics analysis of the samples, excited by radiation with λ = 980 nm, when bimolecular recombination can dominate. Excitation with λ = 980 nm (*hv* = 1.27 eV) leads to appearance of electron–hole pairs only in the InGaAs, while GaAs is transparent in the spectral range (1.43 eV at 290 K). In order to give a contribution to the photoconductivity, electron–hole pairs, photoexcited in QWR or QDs, have to escape from the InGaAs potential well in various ways, such as thermal emission, Poole-Frenkel emission [22, 23], or tunneling [24]. Whereas the activation energy of holes in InGaAs is much smaller than the activation energy of electrons, interband excitation leads to the accumulation of electrons in QWR or QDs and the creation of local electric fields around them. Nanoscale potential barriers around InGaAs/GaAs interfaces significantly reduce the electron capture in InGaAs from GaAs matrix and enhance photoconductivity [25]. This effect is known for low-dimensional InGaAs/GaAs heterostructures as artificial doping effect [26]. Thus, while studying photoconductivity relaxation, excited by interband transitions in InGaAs, we should take into account that the accumulation of negative charges increases the recombination probability of electrons and holes via the QWR quantum states, which leads to non-exponential relaxation described by Eq. (2). Moreover, faster photoconductivity relaxation is because these structures show a higher electron–hole recombination probability via quantum states of InGaAs QWRs compared to QDs. In structure with QWR, linear recombination involving Shockley–Read centers in the GaAs surrounding appeared as dominant channel of photoconductivity relaxation.

## Conclusions

The photoconductivity relaxation was observed at excitation of 650 nm for heterostructures with InGaAs QWR or QD and was described by a stretched exponential function, typical for systems with disorder-induced random energy distribution of traps and distances between localized states. Relaxation appeared slower for structures with QWR, indicating a longer lifetime for charge carriers photoexcited in the GaAs surrounding of InGaAs.

At excitation λ = 980 nm, when photogeneration of electron–hole pairs was only in InGaAs nanoscale objects, the time dependence of photoconductivity is described by kinetic equation that takes into account the linear recombination involving Shockley–Read centers in the GaAs surrounding and bimolecular recombination via quantum-sized states of the InGaAs QWRs. Faster photoconductivity relaxation in the structures with QWR, compared with quantum dots, indicates a higher probability of electron–hole recombination.
